# Exosomes from cisplatin-induced dormant cancer cells facilitate the formation of premetastatic niche in bone marrow through activating glycolysis of BMSCs

**DOI:** 10.3389/fonc.2022.922465

**Published:** 2022-12-09

**Authors:** Jiaqi Xu, Xiang Feng, Na Yin, Lujuan Wang, Yaohuan Xie, Yawen Gao, Juanjuan Xiang

**Affiliations:** ^1^ Hunan Cancer Hospital and the Affiliated Cancer Hospital of Xiangya School of Medicine, Central South University, Changsha, Hunan, China; ^2^ Cancer Research Institute, School of Basic Medical Science, Central South University, Changsha, Hunan, China; ^3^ The Key Laboratory of Carcinogenesis of National Health Committee and the Key Laboratory of Carcinogenesis and Cancer Invasion of the Chinese Ministry of Education, Xiangya Hospital, Central South University, Changsha, Hunan, China; ^4^ Hunan Key Laboratory of Nonresolving Inflammation and Cancer, Changsha, Hunan, China; ^5^ Department of Oncology, The Second Xiangya Hospital of Central South University, Changsha, China; ^6^ Hunan Key laboratory of Early Diagnosis and Precise Treatment of Lung Cancer, Department of Thoracic Surgery, the Second Xiangya Hospital, Central South University, Changsha, Hunan, China

**Keywords:** BMSCs, exosomes, premetastatic niche, IGF-1R, glycolysis

## Abstract

**Introduction:**

Lung cancer is the leading cause of cancer-related deaths worldwide. Chemotherapy kills most cancer cells; however, residual cells enter a dormant state. The dormant cancer cells can be reactivated under specific circumstances. The “premetastatic niche” that is suitable for colonization of cancer cells is formed before the arrival of cancer cells. Tumor-derived exosomes are the main mediators of tumorigenesis. We are aiming to elucidate the roles of exosomes from cisplatin-induced dormant lung cancer cells in the formation of premetastatic niches in bone marrow.

**Methods:**

We performed differential proteomics in dormant A549 cell- and A549 cell-derived exosomes. Non-targeted metabolomics and RNA sequencing were performed to explore the molecular and metabolic reprogramming of bone marrow stromal cells (BMSCs). The growth and metastasis of A549 cells *in vivo* were monitored by bioluminescence imaging.

**Results:**

We found that Insulin-like growth factor 2 (IGF-2) and Insulin-like growth factor binding protein 2 (IGFBP2) were upregulated in dormant A549 cell-derived exosomes. BMSCs that took up exosomes from dormant A549 cells showed enhanced glycolysis and promoted the growth and metastasis of A549 cells possibly through Insulin-like growth factor 1 receptor (IGF-1R)-induced metabolic reprogramming. Inhibition of the production of lactate and IGF-1R signaling can suppress the growth and metastasis of A549 cells from bone marrow.

**Discussion:**

Overall, we demonstrated that BMSCs formed a premetastatic niche upon taking up exosomes from cisplatin-induced dormant lung cancer cells. BMSCs promoted lung cancer cell growth and metastasis through the reverse Warburg effect.

## Introduction

Lung cancer is the most common malignant cancer in terms of both incidence and mortality (1). Cisplatin-based chemotherapy is a first-line treatment for non-small cell lung cancer (NSCLC) patients. Chemotherapeutic drugs can kill most of the dividing cancer cells, but there may be a small number of residual cancer cells that are latent in the body in a temporary dormant stage and difficult to be detected (2). The dormant cancer cells may reenter the proliferative stage, leading to cancer recurrence and metastasis (3). The residual dormant cancer cells share the characteristics of cancer stem cells that are of high mobility and drug resistance (4). Lung cancer cells have a particular affinity to proliferate in bones. Bone marrow is a sanctuary site for dormant disseminated tumor cells (DTCs) (5). Distant recurrences arise from DTCs that lie dormant (6). The reciprocal interaction between the bone marrow microenvironment and cancer cell may decide the proliferation or growth arrest of cancer cells (3). Recent studies demonstrated the changes occurring in distant tissues that prime the microenvironment to adapt incoming cancer cells, which is called “premetastatic niche” (7). The tumor stroma evolves during the primary tumor development (8). The bone marrow microenvironment is the niche for dormant cancer cells (9). It is well known that bone marrow stromal cells (BMSCs) are the main components of the bone marrow microenvironment.

Exosomes are small extracellular carriers that are released by cells into the extracellular environment, carrying various types of molecules such as DNA, messenger RNA (mRNA), noncoding RNA, lipids, and proteins (10, 11). Secreted factors including exosomes are responsible for the premetastatic niche formation (12).

In this study, the effect of dormant lung cancer cell-derived exosomes on the formation of the premetastatic niche in bone marrow was investigated. We found that dormant lung cancer cell-derived exosomes can be taken up by BMSCs. As an important component of the bone marrow microenvironment, BMSCs promoted lung cancer cell growth in the bone marrow and following second dissemination. Exosomes from dormant lung cancer cells enhanced the glycolysis of BMSCs through IGF-1R signaling. Inhibition of IGF-1R or glycolysis can reduce the lung cancer cell growth in the bone marrow.

## Materials and methods

### Cells

Human BMSCs were obtained from bone marrow aspirates of non-hematologic malignant tumor patients. The isolation and culture of BMSCs were performed using methods described previously (13–15). Samples were from Xiangya Hospital, Central South University, China. The collection of the bone marrow was performed for diagnosis. The patients were informed about the sample collection and have signed informed consent forms. Collections and use of tissue samples were approved by the ethical review committees of Xiangya Hospital. Bone marrow aspirates were collected and stored in evacuated tubes containing anticoagulants. Mononuclear cells were isolated from the bone marrow using Ficoll-PaqueTM PLUS (Density 1.077 ± 0.001 g/ml, GE Healthcare, 17144002). The mononuclear cells were collected and cultured in flasks. Three days after plating, the non-adherent cells were removed. When the adherent cells reached confluence, the cells were then passaged and used for the following experiments. Human BMSCs are a monolayer cultured in Human Mesenchymal Stem Cell Growth Medium (HUXMA-03011-440, cyagen).

A short-term single dose of cisplatin was used to induce the dormant A549 cells. Briefly, A549 cells were plated into 10-cm dishes and cultured in the RPMI 1640 (SH30809.01B, Hyclone, USA) complete medium (including 10% serum) (04–001-1ACS, BI, Israel). When cells grew at about 50%–60% confluence, the medium was changed. Cisplatin was added into the medium at a final concentration of 7 ng/μl. After treatment for 48 h, fresh medium was changed and replaced every 2 days. After 16 days of culture, the dormant A549 cells were collected and used for subsequent research. Cell cycle was used to evaluate the dormant cell model.

### Extraction of exosomes

A549 cells were cultured in the RPMI 1640 complete medium (including 10% serum). When cells grew at about 50%–60% confluence, the medium was changed to RPMI 1640 with exosome-free serum. The exosome-free serum was produced by ultracentrifugation at 1 × 10^5^ g (Beckman Coulter Avanti J-30I, USA) at 4°C for 16 h to remove the exosome in the serum, followed by purifying with 0.22-µm filter (Millipore, USA). After replacing the medium, A549 cells were cultured for an additional 48 h and the medium was collected for exosome isolation. In addition, after cisplatin treatment for 2 days, the residual A549 cells were cultured for an additional 16 days in exosome-free serum until the cells regrew. The medium was collected again for exosome isolation for the cells in the reactivated state. The medium was separated by differential centrifugation of 300 g for 10 min, 2,000 g for 15 min, and 12,000 g for 30 min to remove floating cells and cellular debris. The medium was then centrifuged at 4,000 g for 1 h and concentrated to 0.5 ml at 4°C after passing through a filter of 0.22 µm. Finally, the EXO Quick-TC™ exosome isolation reagent (EXOTC50A-1, System Biosciences, USA) was added to the concentrated solution at a ratio of 1:5. After mixing well, the product was then left to stand still at 4°C for 12 h, followed by centrifuging at 1,500 g for 30 min at 4°C. The supernatant was discarded, and the precipitation was resuspended with 20–100 µl phosphate buffered saline (PBS).

### Transmission electron microscopy

Extracted exosomes were fixed in 1% dialdehyde for 10 min and washed with deionized water. In this study, 10 µl of exosome suspension was placed on formvar carbon-coated 300-mesh copper electron microscopy grids (Agar Scientific Ltd., Stansted, UK) and incubated at room temperature for 5 min. Then, the exosomes were negatively stained with 2% uranyl oxalate for 1 min at room temperature and followed by washing three times with PBS and drying at room temperature. Images were obtained by microscopy [transmission electron microscopy (TEM)] (JEM-2100, Quan Luo, Japan).

### Size of the analyzed exosomes

Exosomes were resuspended in 1 ml PBS and analyzed using NANO ZS 90 (Zetasizer Nano ZS90 Instrument, Malvern, UK) according to the manufacturer. All samples were measured with parameters of 44.5 mm and 0.64 V voltage using NP100 membranes. Data were analyzed by Zetasizer software (Malvern Instruments) that was optimized to identify and track each particle on a frame-by-frame basis.

### Exosome tracing

PKH67 (MINI67-1KT, Sigma, USA) was used to label the exosomes according to the previous research (15). Briefly, exosomes were collected and resuspended in 500μl Diluent C mixed with 4 μl PKH67. Exosome-free fetal bovine serum (FBS) was added into the exosome suspension to stop the reaction and remove the excess PKH67. The labeled exosomes were washed and resuspended in PBS. BMSCs were plated on coverslips in six-well plates. When the cells reached 80% confluence, the labeled exosomes were added in culture media and were incubated with BMSCs for 6 h. The cells were washed twice with PBS and fixed with 4% paraformaldehyde for 30 min at room temperature. 4',6-diamidino-2-phenylindole (DAPI, D9542, Sigma, USA) was used to stain the cell nucleus. After staining, the glass coverslips were washed twice with PBS and observed by fluorescence microscope (BX53, Olympus, Japan).

### BMSC incubation with exosomes

BMSCs were inoculated into six-well plates and cultured with exosome-free medium for 24 h before treatment. When the cell confluence reached 70%–80%, exosomes (200 μg) were added into the culture medium per well. The control group was treated with PBS. After 48-h treatment, BMSCs were collected for the subsequent experiments. BMS-536924 (IGF-1R inhibitor, Selleck, USA) at 10 μM was applied to inhibit the IGF pathway. Moreover, GSK (GSK2837808A, lactate dehydrogenase A inhibitor, MedChemExpress, USA) at 75 μM was applied to inhibit glycolysis. BMS-536924 and GSK2837808A were both added in exosome-treated BMSCs 48 h after exosome treatment.

### Cell proliferation assay

Before measuring the proliferation rate of A549 cells, BMSCs treated with exosomes were cocultured with A549 cells by using the six-well Transwell system (3412, Corning, USA). BMSCs were incubated with exosomes and inoculated into the upper chamber at a density of 5 × 10^5^ cells per well. A549 cells were inoculated into the upper chamber at a density of 2 × 10^5^ cells per well. RPMI 1640 medium with 10% FBS was added into the coculture systems and maintained for 48 h. After coculturing, A549 cells were collected and inoculated into 96-well at a density of 1 × 10^3^ cells per well. Cell Counting Kit-8 (CCK8, A311-01, Vazyme, China) was used to measure the proliferation rate of A549 cells after coculturing with BMSCs treated with exosomes.

### Western blotting

MSCs were plated in six-well plates at a density of 2 × 10^5^ cells/well for 24 h and treated with exosomes at a concentration of 200 µg for 48 h. After removing the medium, cells were lysed in a buffer (P0013 and 1 mM phenylmethylsulfonyl fluoride (PMSF), Beyotime Institute of Biotechnology, Shanghai, China) for 30 min at 4°C. Exosome sample preparation was similar as before. The supernatant was harvested by centrifuging at 12,000 g for 5 min at 4°C. A bicinchoninic acid (BCA) kit was used to measure protein concentrations. The proteins were separated by 10% sodium dodecyl sulphate-polyacrylamide gel electrophoresis (SDS-PAGE) and transferred to nitrocellulose membranes. The membranes were blocked with 5% bovine serum albumin (BSA) for 1 h, detected for anti-HSP70 (EXOAB-Hsp70A-1, System Biosciences, USA), anti-CD63 (EXOAB-CD63A-1, System Biosciences, USA), anti-TSG101 (28283-1-AP, Proteintech, USA), anti-IGFBP2 (11065-3-AP, Proteintech, USA), anti-IGF-2 (ab177467, Abcam, UK), anti-IGF-1R (9750T, Cell Signaling Technology, USA), and anti-p-IGF-1R (Tyr1135, 3918T, Cell Signaling Technology, USA) at 4°C overnight, and then incubated with anti-rabbit IgG conjugated with horseradish peroxidase (HRP) (SA00001-2, Proteintech, USA) for 1 h at room temperature. The Infrared Imaging System (LI-COR Biosciences, Lincoln, NE, USA) was used to scan and analyze the images.

### Multiplexed tandem mass tag labeling coupled with two-dimensional liquid chromatography tandem mass spectrometry (TMT-LC/LC-MS/MS)

Exosomes were sonicated three times on ice using a high-intensity ultrasonic processor (Scientz) in lysis buffer (8 M urea, 1% Protease Inhibitor Cocktail). The remaining debris was removed by centrifugation at 12,000 g at 4°C for 10 min. Finally, the supernatant was collected, and the protein concentration was determined with a BCA kit according to the manufacturer’s instructions. The protein samples were diluted by adding 100 mM triethylammonium bicarbonate (TEAB) to urea concentration. Filter-aided sample preparation (FASP) was used to remove impurities. Trypsin was then added at 1:50 trypsin-to-protein mass ratio for 12 h. The tryptic peptides were dissolved in 0.5 M TEAB. Each channel of peptide was labeled with its respective tandem mass tag (TMT) reagent (according to the manufacturer’s protocol; ThermoFisher Scientific) and incubated for 2 h at room temperature. A specific class of isobaric labels is composed of a mass reporter region, a cleavable linker region, a mass normalization region, and an amine-reactive group. In multiplex TMT labeling, peptides from individual samples are tagged with a TMT labeling reagent with a unique reporter ion. The samples were desalted with Strata X C18 SPE column (Phenomenex) and dried using vacuum centrifugation.

The tryptic peptides were dissolved in 0.1% formic acid (solvent A) directly loaded onto a homemade reversed-phase analytical column (15-cm length, 75 μm i.d.). The gradient was composed of an increase from 6% to 23% solvent B (0.1% formic acid in 98% acetonitrile) over 26 min, 23% to 35% in 8 min, and climbing to 80% in 3 min, then holding at 80% for the last 3 min, all at a constant flow rate of 400 nl/min on an EASY-nLC 1000 ultra performance liquid chromatography (UPLC) UPLC system.

The peptides were subjected to an nanospray ionization (NSI) source followed by tandem mass spectrometry (MS/MS) in Q ExactiveTM Plus (Thermo) coupled online to the UPLC. The electrospray voltage applied was 2.0 kV. The m/z scan range was 350 to 1,800 for full scan, and intact peptides were detected in the Orbitrap at a resolution of 70,000. Peptides were then selected for MS/MS using Normalized Collision Energy (NCE) setting of 28, and the fragments were detected in the Orbitrap at a resolution of 17,500. A data-dependent procedure that alternated between 1 MS scan and 20 MS/MS scans with 15.0s dynamic exclusion. Automatic gain control (AGC) was set at 5E4. Fixed first mass was set at 100 m/z. The resulting MS/MS data were processed using Maxquant search engine (v.1.5.2.8). The original data were deposited in iProX database with accession number IPX0005220000.

### Metabolomics

BMSCs incubated with exosome and corresponding culture medium were collected for metabolomics. After the sample was slowly thawed at 4°C, we got an appropriate amount of sample and added it to the precooled methanol/acetonitrile/water solution (2:2:1, v/v); vortex to mix; sonicate at low temperature for 30 min. The samples were let to stand at -20°C for 10 min, centrifuged at 14,000 g at 4°C for 20 min. The supernatant was collected and vacuum dried. For mass spectrometry analysis, 100 μl of acetonitrile aqueous solution (acetonitrile:water = 1:1, v/v) was added to reconstitute, vortex, and centrifuge at 14,000 g at 4°C for 15 min. The supernatant was recollected for analysis. The samples were separated using Agilent 1290 Infinity LC Ultra High-Performance Liquid Chromatography (UHPLC) System HILIC column. Then, the AB Triple TOF 6600 mass spectrometer was used to collect the primary and secondary spectra of the samples. Finally, data analysis was performed. The original data were deposited in MetaboLights database with accession number MTBLS6135.

### RNA sequencing platform technologies and pipelines

Total RNA was extracted from the cells using TRIzol (Invitrogen, CA, USA) according to manual instruction. Total RNA was qualified and quantified using a NanoDrop and Agilent 2100 bioanalyzer (Thermo Fisher Scientific, MA, USA). Total RNA was isolated from BMSCs, and the cDNA library was constructed. Oligo(dT)-attached magnetic beads were used to purify mRNA. Purified mRNA was fragmented into small pieces with fragmentation buffer. Random hexamer-primed reverse transcription was performed to synthesize double-stranded cDNA. The cDNA fragments were amplified by PCR, and products were purified by Ampure XP Beads. The product was validated on the Agilent Technologies 2100 bioanalyzer for quality control. The final library was sequenced with Illumina platform (Applied Protein Technology, China). The clean reads were mapped to the reference genome (Homo_sapiens, GCF_000001405.38_GRCh38.p12) using HISAT2 v2.0.4) (16, 17). The expression levels for each of the genes were normalized as fragments per kilobase of exon model per million mapped reads (FPKM) by RNA Sequencing (RNA-Seq) Expectation Maximization (RSEM). Essentially, differential expression analysis was performed using the DESeq2 (v1.4.5) (18) with P value ≤0.05. Gene Ontology enrichment and Kyoto Encyclopedia of Genes and Genomes (KEGG) enrichment were performed with a Fisher’s exact test. The raw RNA-seq data were deposited in the Sequence Read Archive (SRA) database with accession number SRA PRJNA890755.

### Assay for lactic acid

Lactic acid concentration was quantified by corresponding assay kits (KTB1100, Abbkine, China) according to the manufacturer’s instructions. Briefly, BMSCs incubated with exosomes were collected. About 5 × 10^6^ cells per group were used for the next step. The cells were washed twice with PBS and resuspended with 500 μl Lactate Assay Buffer. BMSCs were broken by sonication and then centrifuged at 12,000 g for 5 min at 4°C. The supernatant was collected. The working fluid per well (55 μl) was prepared with 31 μl Lactate Assay Buffer, 8 μl Lactate Dehydrogenase Cofactor, 5 µl water-soluble tetrazolium-8 (WST-8), 1 μl Enhancer, and 10 μl lactate dehydrogenase. The supernatant and standard samples were added to each well. After that, the working fluid was added to each well and incubated for 30 min at 37°C. The absorbance at 450 nm was measured by Varioskan LUX multifunctional reader (ThermoFisher, USA).

### Glycolysis stress assay

The glycolytic function of BMSCs was assessed using Extracellular Flux Analyzer (SeaHorse Bioscience, MA). The Seahorse glycolytic stress test was carried out according to the manufacturer’s instructions (Agilent Seahorse Bioscience). Briefly, cells were plated into cell culture plates. The cells were incubated for 24 h in a humidified 37°C incubator with 5% CO_2_ to allow them to adhere to the plates. For the glycolysis stress test, 2.5 ml each of 250 mM glucose, 10 µM oligomycin, and 500 mM 2-deoxyglucose (2-DG) in glycolysis stress assay medium was prepared. The compounds were loaded into the appropriate injector ports of the cartridge, and the program was run.

### Animal experimental protocols

Female nude mice aged 4–6 weeks were randomly divided into seven groups, with five mice each group. The first group was inoculated with BMSCs which were treated with PBS, and the second group was inoculated with BMSCs which were incubated with exosomes derived from A549 cells. The third group was inoculated with BMSCs which were incubated with exosomes derived from cisplatin-induced dormant A549 cells. BMS-536924 (IGF-1R inhibitor, Selleck, USA) and GSK (GSK2837808A, lactate dehydrogenase A inhibitor, MedChemExpress, USA) were also applied in this experiment as described in section BMSCs incubated with exosomes. The bone marrow cavity of mice was injected with 10^4^ of A549 cell-derived exosome (exo-) or dormant A549 cell-derived exosome (DORexo)-treated BMSCs. Two days after injection of BMSCs or PBS, 10 µl of lung cancer cells expressing luciferase A549 (2 × 10^6^/ml) were injected into the mouse bone marrow cavity to prepare a mouse model. The experimental procedure was amended according to previously published protocols (19). Mice were anesthetized with isoflurane. The knee of the mouse was flexed beyond 90 degrees. The surface of the tibia articular was exposed to make a hole for tumor cell injection. The cancer cells were injected from the knee joint to the medullary cavity of the tibia. On the sixth day, the mice were anesthetized by isoflurane inhalation, and substrate D-luciferin was intraperitoneally injected into mice, and then the fluorescence intensity of cancer cells in the mice was monitored by bioluminescence imaging (IVIS Lumina LT Series III, PerkinElmer, USA). The changes of fluorescence signal in mice were observed dynamically.

### Statistical analysis

All statistical analyses were performed using the SPSS 20.0 software, and the graphs were generated using GraphPad Prism 8.0 (GraphPad Software, San Diego, CA, USA). The comparisons of means among groups were analyzed by one-way ANOVA, and the Dunn multiple comparison test was further used to determine significant differences between groups.

## Results

### Exosomes derived from cisplatin-induced dormant A549 cells promote the growth and metastasis of cancer cells in the bone marrow by educated BMSCs

**Figure 1:**
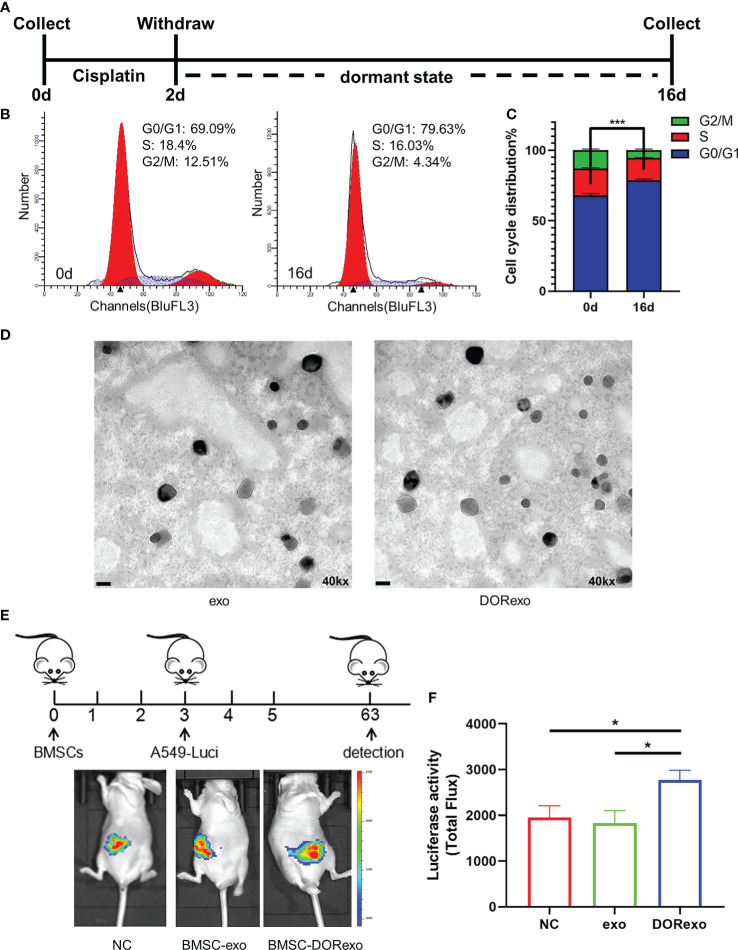
Exosomes derived from cisplatin-induced dormant A549 cells promote the growth and metastasis of cancer cells in the bone marrow. **(A)** Schematic diagram of the cisplatin treatment experiment. A549 cells were treated with cisplatin for 2 days. The remaining cells after treatment were cultured for an additional 14 days. **(B)** Cell cycle analysis of cisplatin-treated A549 cells. **(C)** The ratio of cells with different cell cycles was represented in the histogram graph. G0–G1 phase, ***P < 0.001. **(D)** Transmission electron microscopy images of exosomes isolated from cancer cells. exo, exosomes from untreated A549 cells; DORexo, exosomes from dormant A549 cells. Scale bar: 100 nm. Magnification: ×40,000. **(E)** Schematic diagram of animal experiments. BMSCs that were incubated with exosomes were injected into the bone marrow cavity, and A549-luciferase cells were injected into the bone marrow cavity 3 days later. The growth and metastasis A549 were evaluated by bioluminescence. All mice were intramedullary injected with luciferase-labeled A549 cells. Animal imaging fluorescence images: the imaging image on 63 days after A549-luciferase cells’ injection. NC, mice without BMSC injection; BMSC-exo, mice with injection of exo-treated BMSCs; BMSC-DORexo, mice with injection of DORexo-treated BMSCs. **(F)** The activities of luciferase were shown in the histogram. *P < 0.05.

In order to determine the effect of dormant cancer cell-derived exosomes in carcinogenesis, we isolated and characterized exosomes from cisplatin-induced dormant A549 cells and intact A549 cells. In this study, we introduced an *in vitro* model of tumor dormancy induced by short-term treatment with cisplatin according to previously published protocols (20–22). We firstly treated lung adenocarcinoma cell line A549 with cisplatin at 7 ng/µl. Two days after treatment, cisplatin was withdrawn and the medium was changed. The remaining cells continued to be cultured for an additional 14 days (**Figure 1A**). Most cells were killed, and the residual cells entered into G0–G1 phase (P < 0.001; **Figures 1B, C**), which indicated that the cells were in a dormant state. We then collected exosomes from untreated A549 cells (exo) and dormant A549 cells (DORexo). The exosomes were observed by TEM (**Figure 1D**). The exosomes were positive for exosomal markers HSP70, CD63, and TSG101 (**Supplementary Figure S1**). Size analysis using NANO ZS 90 revealed that the exosomes collected from dormant A549 cell media were bigger than those from untreated A549 cell media, with average diameters of 90 and 40 nm, respectively (**Supplementary Figure S2**).

Given the key role of BMSCs in the bone marrow microenvironment, we investigated whether DORexo could educate BMSCs and affect cancer cell growth in the bone marrow. We incubated BMSCs *in vitro* with exo and DORexo. The exosomes were labeled with fluorescence PKH67. As shown in **Supplementary Figure S3**, the fluorescent exosomes entered the BMSCs after 6-h incubation. We then injected exosome-treated BMSCs (exo BMSCs and DORexo BMSCs) into the bone marrow of nude mice, followed by injection of luciferase-positive A549 in the bone marrow 3 days later (**Figure 1E**). The growth and metastasis of cancer cells *in vivo* were monitored by bioluminescence imaging. As shown in **Figure 1E**, the bioluminescent signals were mostly observed in the abdomen, indicating the spread of A549 from the bone marrow. The DORexo BMSCs enhanced the cancer cell metastasis in the nude mice compared to mice that received injections of untreated BMSCs and the exo BMSCs (**Figures 1E, F**). Although the signals were absent in the bone marrow, we speculated that cancer cells inoculated in the bone marrow determine the metastatic growth in the abdomen. It indicated that exo can influence the growth in the bone marrow and metastasis of cancer cell from the bone marrow by educating BMSCs.

### Proteomic analysis of the differential protein in exosomes

**Figure 2:**
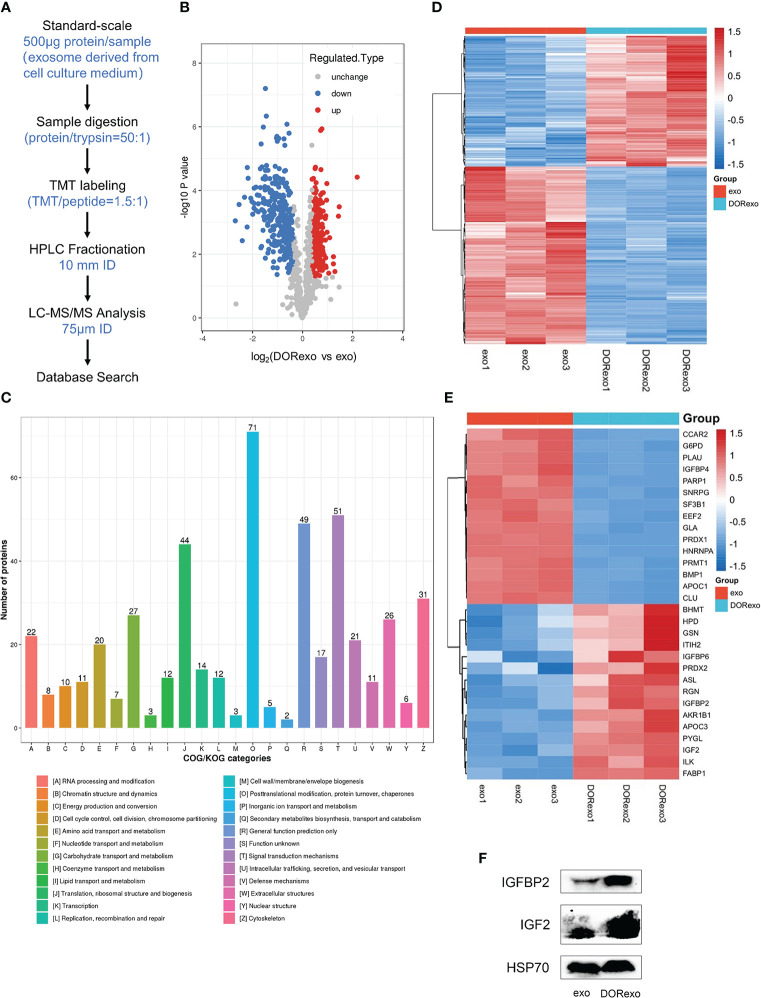
Proteomic analysis of the differential protein in dormant A549 cell exosomes. **(A)** The analysis pipeline of proteomics was shown. **(B)** The volcano plot was shown. Red dots indicate expressed upregulated proteins, and blue dots indicate expressed downregulated proteins. **(C)** Gene Ontology was performed on differentially expressed exosome proteins. The differentially expressed proteins were enriched in the Clusters of Orthologous Groups of protein/EuKaryotic Orthologous Groups (COG/KOG) categories. **(D)** Heat map showed the enrichment of 394 proteins involved in metabolic processes. **(E)** Heat map showed the enrichment of proteins involved in the cellular metabolic processes and in the IGF signaling pathway. **(F)** IGFBP2 and IGF-2 were analyzed by Western blotting exo, exosomes from untreated A549 cells; DORexo, exosomes from dormant A549 cells.

We measured the differential proteome in exo and DORexo by proteomic analysis. The proteomic detection of exosomes was performed by TMT labeling coupled with nanoscale liquid chromatography tandem mass spectrometry (LC-MS/MS) analysis (**Figure 2A**). In this study, a total of 996 proteins were quantified that were jointly found in two samples (**Supplementary Figure S4**). The differentially expressed proteins were obtained by two steps. The average value of each sample was firstly obtained from multiple repeats, and the ratio of the mean values was used for the final quantities. The significance of the difference between two samples was displayed as P-value. The difference multiple (log2 value) and P-value (-log10 value) were used as the abscissa and ordinate to generate the difference protein volcano map. The proteins with a 1.3-fold differential expression change were identified, and a statistical t-test with a P-value <0.05 was considered to be significant. A total of 265 proteins were found upregulated and 313 proteins were found downregulated in the DORexo compared to the exo (**Figure 2B**). The differentially expressed proteins were enriched in the EuKaryotic Orthologous Groups (KOG) classification of Carbohydrate transport and metabolism, Lipid transport and metabolism, etc. (**Figure 2C**). Heatmaps were generated for these 394 differentially expressed proteins related to the metabolic process (**Figure 2D**). The differentially expressed proteins with the most fold change value including IGF-2 and IGFBP2 were shown in **Figure 2E**. We confirmed the expressions of IGF-2 and IGFBP2 in exosomes by Western blotting assays (**Figure 2F**; **Supplementary Figure S5**).

### Exosomes derived from cisplatin-induced dormant A549 cells promote cancer cell growth by bone marrow education through IGF-1R

**Figure 3:**
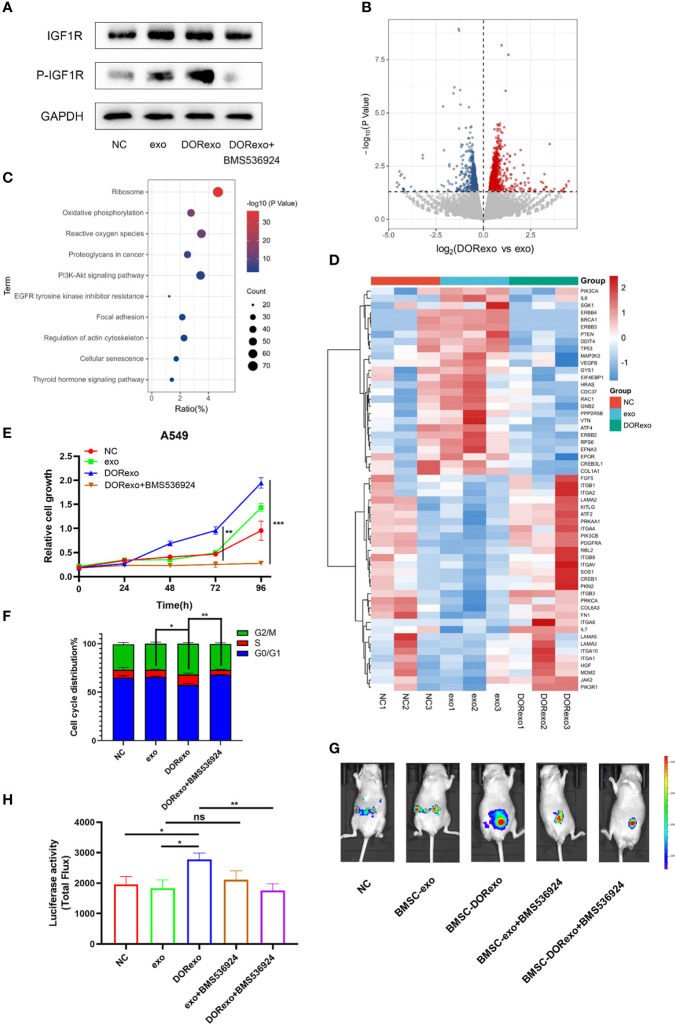
Exosomes derived from cisplatin-induced dormant A549 cells promote cancer cell growth by activating the BMSC IGF pathway. **(A)** Protein expressions of IGF-1R, p-IGF-1R were measured by Western blot analysis. **(B)** The volcano plots show differentially expressed genes (DEGs) between exo-treated BMSCs and DORexo-treated BMSCs based on RNA-seq (P < 0.05). **(C)** The bubble diagram showed KEGG pathway enrichment analysis for DEGs (0.5-fold, P < 0.05). **(D)** The heat map showed the significant differences involved in phosphoinositide 3-kinase (PI3K)-Akt signaling pathway and focal adhesion-related genes. **(E)** Cell viability of A549 cocultured with BMSCs treated with different exosomes and BMS-536924. **(F)** The ratio of cells with different cell cycles was represented in the histogram graph. S phase, *P < 0.05, **P < 0.01. **(G)** Animal imaging fluorescence images. BMSCs that were incubated with exosomes were injected into the bone marrow cavity, and A549-luciferase cells were injected into the bone marrow cavity 3 days later. The growth and metastasis of A549 were evaluated by bioluminescence. IGF-1R inhibitor BMS-536924 was applied in the experiment. All mice were intramedullary injected with luciferase-labeled A549 cells. NC, mice without BMSC injection; MSC-exo, mice with injection of exo-treated BMSCs; MSC-DORexo, mice with injection of DORexo-treated BMSCs; MSC-exo+BMS-536924, mice with injection of exo-treated BMSCs and BMS-536924; MSC-DORexo+BMS-536924, mice with injection of DORexo-treated BMSCs and BMS-536924. **(H)** The activities of luciferase were shown in the histogram. *P < 0.05; **P < 0.01; ***P < 0.001. ns, no significance.

As shown above, DORexo contained enhanced levels of IGF-2 and IGFBP2, and we are wondering if the promotive effect of BMSCs on cancer cell growth in bone marrow is through activating IGF-1R. After being incubated with 200 μg of DORexo, BMSCs showed increased IGF-1R expression and phosphorylated IGF-1R (**Figure 3A**; **Supplementary Figure S6**). In order to validate the activating effect of DORexo in IGF-1R, we added IGF-1R inhibitor BMS-536924 in DORexo-treated BMSCs 48 h after DORexo treatment. The addition of BMS-536924 in DORexo-treated BMSCs decreased the expression of phosphorylated IGF-1R (**Figure 3A**). It is suggested that DORexo activate the IGF pathway in BMSCs. To investigate the differential gene expression profiling between the BMSCs treated with exosomes, we performed RNA-seq on BMSCs treated with exo and DORexo. There were 1,735 differentially expressed genes (DEGs) between exo-treated BMSCs and DORexo-treated BMSCs including 1,011 upregulated and 724 downregulated genes (P < 0.05, **Figure 3B**). All of the DEGs were subjected to KEGG pathway enrichment analysis. As shown in **Figure 3C**, pathway enrichment with significant difference (P < 0.05) indicated that DEGs included the genes related to phosphoinositide 3-kinase (PI3K)-Akt signaling pathway, focal adhesion (**Figure 3C**). The heatmap revealed that the most significant differences involved the PI3K-Akt signaling pathway and focal adhesion-related genes *ITGA*, *ITGB*, etc. (**Figure 3D**). Although there was a lack of enhanced expression of IGF-2 and IGFBP2 at the mRNA level, we assumed that the transfer of IGF-2 and IGFBP2 at the protein level activates the downstream PI3K-Akt signaling. We speculated on the activation of IGF-1R signaling by DORexo. We then added BMS-536924 to cultured DORexo-treated BMSCs. The BMSCs were cocultured with A549 cells, and the proliferation of A549 was evaluated by CCK8 assay. As shown in **Figure 3E**, BMS-536924, an IGF-1R inhibitor, when added into the DORexo-treated BMSCs, could inhibit the promotion of cancer cell proliferation cocultured with the DORexo BMSCs (**Figure 3E**, P < 0.05). The cell cycle analysis revealed that the A549 cells cocultured with DORexo BMSCs had more S phase cells (P < 0.05), and BMS-536924 can reverse the promotive effect (P < 0.01, **Figure 3F**). In order to elucidate the roles of activated BMSCs in cancer cells, we intramedullary injected BMSCs followed by injection of A549-luciferase cells. The growth and metastasis of cancer cells were monitored by bioluminescence imaging. DORexo BMSCs increased cancer cell proliferation compared to untreated BMSCs and exo BMSCs (**Figures 3G, H**). However, we observed a luminescence signal in the abdomen but not in the bone. It suggested metastasis of cancer cells from bone. From **Figures 1E, F**, we found that BMS-536924 treated-exo BMSCs or -DORexo BMSCs displayed reduced metastasis compared to exo BMSCs and DORexo BMSCs. The promotive effect of DORexo-treated BMSCs in metastasis could be reversed by the addition of BMS-536924 in culture media. It implied that DORexo BMSCs increased the survival and metastasis of cancer cells through IGF-1R activation.

### Exosomes promote glycolysis of BMSCs to increase lactic acid production and promote cancer cell growth in the bone marrow

**Figure 4:**
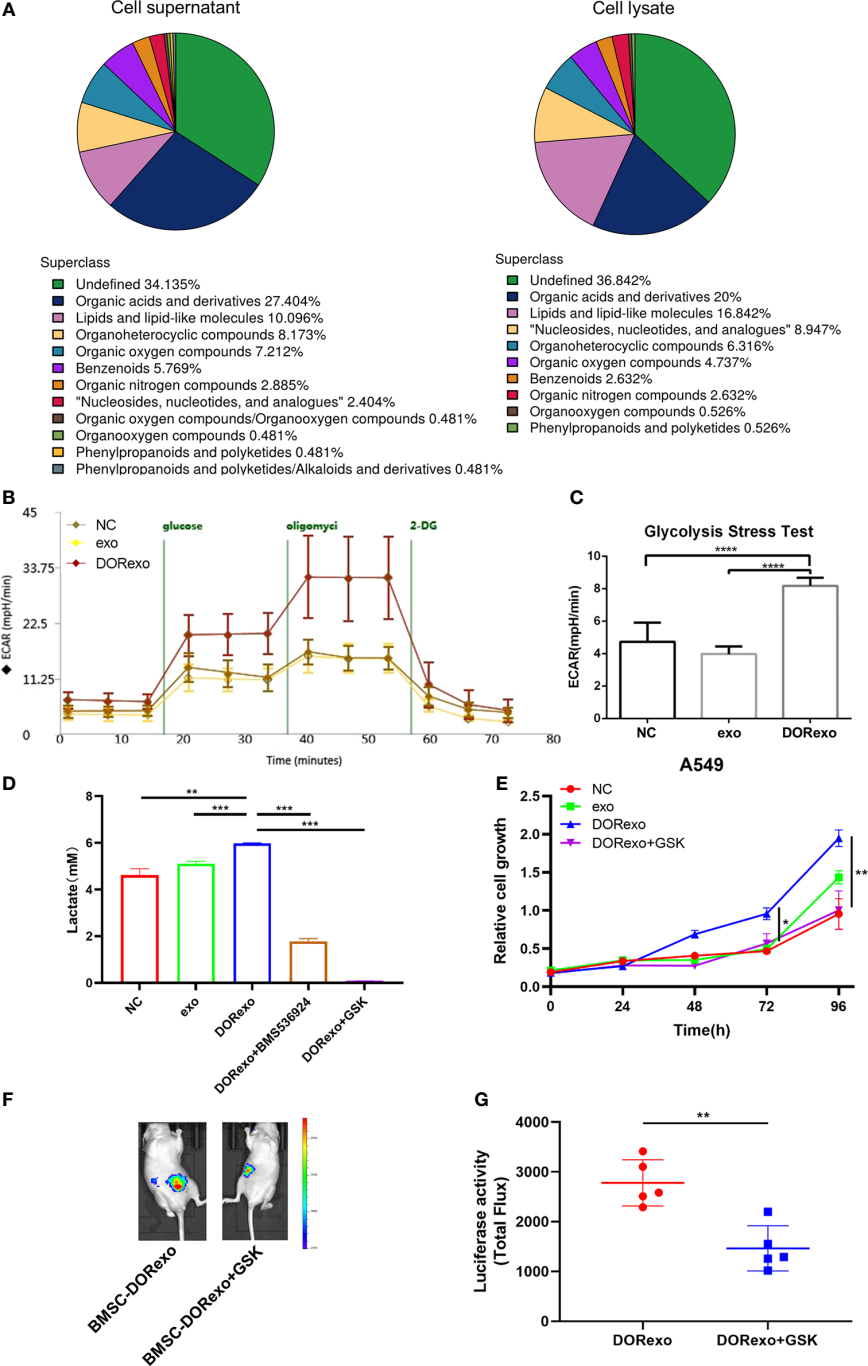
Exosomes promote glycolysis of BMSCs to increase lactic acid production and promote cancer cell growth. Glycolytic stress test was performed to assess the extracellular acidification rates (glycolysis) in BMSCs. **(A)** Metabolite classification in cell lysates and cell supernatants derived from DORexo-treated BMSCs. **(B)** Kinetic ECAR response of exo-treated BMSCs and DORexo-treated BMSCs to glucose, oligomycin, and 2-DG. Each point represents mean ± SD, n  =  16. **(C)** Calculated glycolytic flux, glycolytic capacity. **(D)** Expression of lactic acid in cell supernatants; BMSCs treated with or without GSK were cocultured with A549; BMS-536924, IGF-1R inhibitor; GSK2837808A (GSK), lactate dehydrogenase A inhibitor. **(E)** Proliferative capacity of A549 cocultured with BMSCs treated with different exosomes and GSK. **(F)** Animal imaging fluorescence images on 63 days after A549-luciferase cell injection. BMSCs that were incubated with exosomes were injected into the bone marrow cavity, and A549-luciferase cells were injected into the bone marrow cavity 3 days later. The growth and metastasis A549 were evaluated by bioluminescence. Lactate dehydrogenase A inhibitor GSK2837808A (GSK) was applied in the experiment. All mice were intramedullary injected with luciferase-labeled A549 cells. MSC-DORexo, mice with injection of DORexo-treated BMSCs; MSC-DORexo+GSK, mice with injection of DORexo-treated BMSCs and GSK. **(G)** The activities of luciferase were shown. *P < 0.05; **P < 0.01; ***P < 0.001; ****P < 0.0001..

The reverse Warburg effect emphasizes metabolic reprogramming in stromal cells and the interactions with cancer cells. In order to examine if the metabolic process of BMSCs was altered by DORexo, we performed non-targeted metabolomics in the cell lysate and cell medium of BMSCs. BMSCs were incubated with exosomes. The cell lysates and corresponding culture medium were collected for metabolomics. As shown in **Figure 4A**, organic acids and their derivatives were the main components in both cell lysates and cell supernatants. To further verify whether DORexo affect the glycolytic function of BMSCs, we used the Seahorse XF cell metabolic dynamic analysis system for glycolysis assay. As shown in **Figure 4B**, glucose addition in DORexo-treated BMSCs triggered an increase of extracellular acidification rate (ECAR). The injection of oligomycin, which inhibits the mitochondrial ATP generation, increased the ECAR. The addition of glycolysis inhibitor 2-DG abolished the ECAR. It indicated that exogenous glucose was broken down to lactate, causing an ECAR increase. In contrast, the ECAR values were not significantly different between exo-treated BMSCs and control cells (**Figures 4B, C**). BMSCs that were treated with DORexo produced more lactate than the control group and the exo-treated BMSCs (**Figure 4D**). The addition of GSK2837808, a lactate dehydrogenase A inhibitor, decreased the lactate production by DORexo-treated BMSCs. And the lactate production by DORexo-treated BMSCs was reduced after adding BMS-536924 to inhibit IGF-1R pathway. It indicated that DORexo enhanced the glycolysis of BMSCs through activating IGF-1R (**Figure 4D**). GSK2837808 could inhibit the promotion of cancer cell proliferation induced by the DORexo-treated BMSCs *in vitro* (**Figure 4E**). We then examined the role of inhibition of the production of lactate in the cancer cell growth *in vivo*. We intramedullary injected DORexo-treated BMSCs followed by injection of A549-luciferase cells. The addition of GSK2837808 in the DORexo-treated BMSCs obviously decreased the cancer cell growth *in vivo*, as indicated by bioluminescence (**Figures 4F, G**). It suggested that inhibition of the production of lactate in BMSCs could inhibit the growth and metastasis of lung cancer cells.

## Discussion

In this study, we showed that exosomes derived from chemotherapy-induced dormant cancer cells promoted the premetastatic niche formation and cancer cell survival in the bone marrow. Dormant cancer cell-derived exosomes reprogrammed the metabolic process of BMSCs through transferring exosomal IGF-2 and IGFBP2. The evolving stromal supported the survival of lung cancer cells in the bone marrow through reverse Warburg effects. Our work offers an explanation of the relapse of lung cancer after chemotherapy.

Premetastatic niches are the microenvironment formed before the arrival of cancer cells (9). The nature of the distant microenvironment determines the fate of disseminated cancer cells (23). Exosomes secreted by the primary tumor sites remodel the distant microenvironment and facilitate the formation of premetastatic niches prior to widespread metastasis (24). Comparing the proteins of exosomes from chemotherapy-induced dormant cancer cells and untreated cancer cells, we identified IGF-1R signaling activation as the main mechanism of how cancer cell exosomes educate BMSCs. The transfer of IGF-2 and IGFBP2 from cancer cells to BMSCs enhanced the glycolysis of BMSCs, supporting cancer cell survival in the bone marrow through the production of lactic acid.

The premetastatic niche has characteristics of immunosuppression, inflammation, angiogenesis and vascular permeability, lymph angiogenesis, organotropism, and reprogramming (25). In this study, we found that a high-lactate environment in the bone marrow is detrimental for cancer cell survival and second dissemination from the bone marrow. Lactate has been recognized as an active modulator of the immune response and contributes to the establishment of an immunosuppressive microenvironment (26). Lactate is also a substrate for fatty acid synthesis (27).

Metabolic reprogramming of cancer cells after chemotherapy is a vital hallmark for the occurrence of treatment resistance (28). Aberrant glycolysis and glutaminolysis could promote DNA repair and inhibit the efficacy of chemotherapy (29). Wnt, PI3K/Akt, Notch, Nuclear Factor Kappa B (NF-κB), and mitogenactivated protein kinase (MAPK) signals are implicated in the cellular metabolic stress in chemotherapy resistance (30–32). In this study, chemotherapy-induced lung cancer cells excreted exosomes and transferred IGF-2 into BMSCs. Although there was a lack of enhanced expression of IGF-2 and IGFBP2 at the mRNA level, we speculated that the direct transfer of IGF-2 and IGFBP2 at the protein level activates the downstream pathway. IGF-1R signaling and its downstream MAPK and PI3K/Akt play a major role in tumorigenesis and decrease the tumor latency time (33, 34). Although activation of the IGF-1R signals results in increased glycolysis, proliferative cancer cells take up more glucose than non-proliferating cells (34). The dormant cancer cells may use alternative metabolic mechanisms for survival. In this study, we found that the transfer of exosomal IGF-2 from dormant cancer cells into stromal cells increased the glycolysis of stromal cells. Extracellular lactate excreted from glycolytic stromal cells supported the survival of cancer cells in the bone marrow. Our study reveals a novel mechanism by which exosomes derived from cisplatin-induced dormant cells facilitate the premetastatic niche formation that promotes metastasis.

## Data availability statement

The datasets presented in the study are deposited in the iProX database, accession number IPX0005220000 (https://www.iprox.cn//page/project.html?id=IPX0005220000); MetaboLights database, accession number MTBLS6135 (https://www.ebi.ac.uk/metabolights/index); SRA database, accession number SRAPRJNA890755 (https://www.ncbi.nlm.nih.gov/bioproject/PRJNA890755/).

## Ethics statement

The animal study was reviewed and approved by Biomedical Research Ethics Committee, Hunan Normal University, approval number D2020024.

## Author contributions

JJX and YG conceived the study and applied research projects. JQX and XF focused on the experimental section. NY and LW provided essential technical support. YX contributed to the data analysis. JQX and XF wrote the manuscript. All authors read and approved the final manuscript.
