# Supplementary figures and images for: Exosomes from cisplatin-induced dormant cancer cells facilitate the formation of premetastatic niche in bone marrow through activating glycolysis of BMSCs
Source: Front Oncol. 2022 Dec 9;12:922465. doi: 10.3389/fonc.2022.922465 (PMC9786109; doi:10.3389/fonc.2022.922465)

**Original western blots：**

**1. Figure 1E**

HSP70：


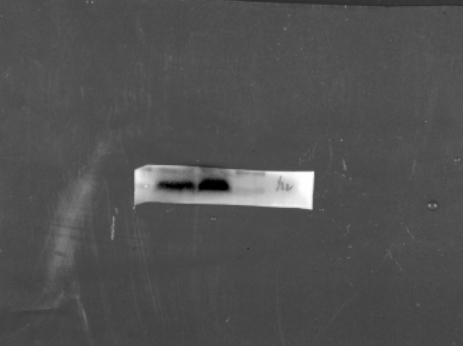

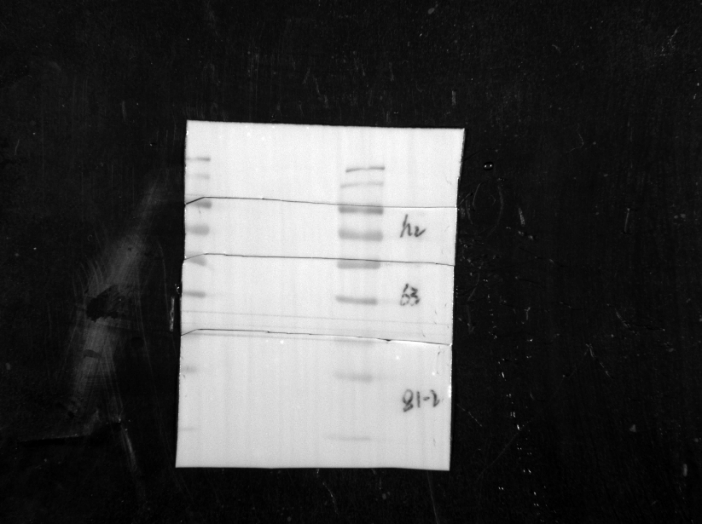


CD63：


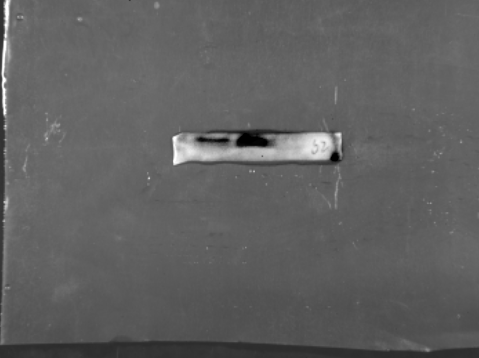

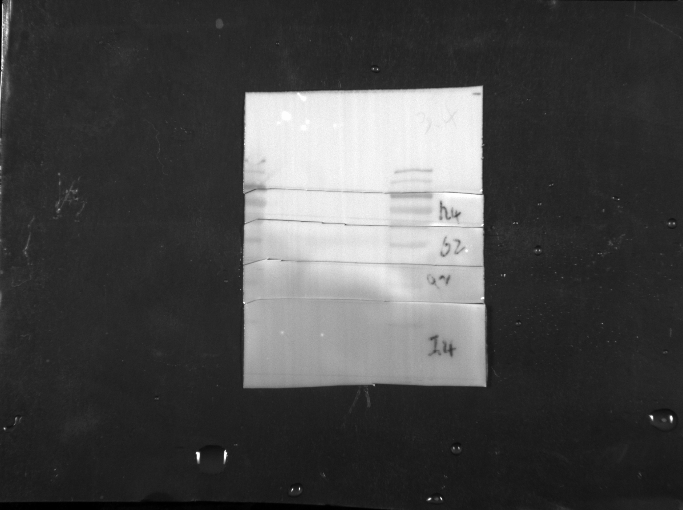


TSG101:


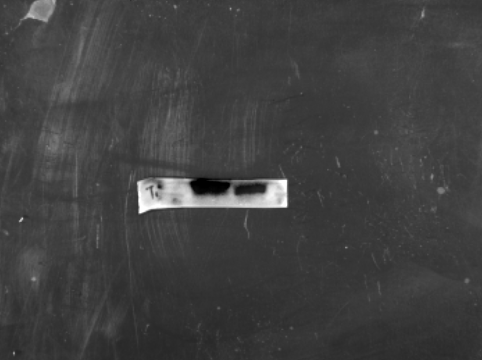

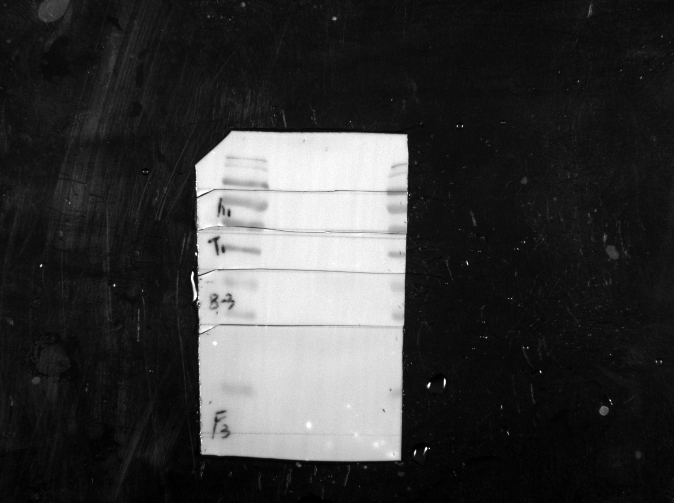


**2. Figure2 D**

IGFBP2：


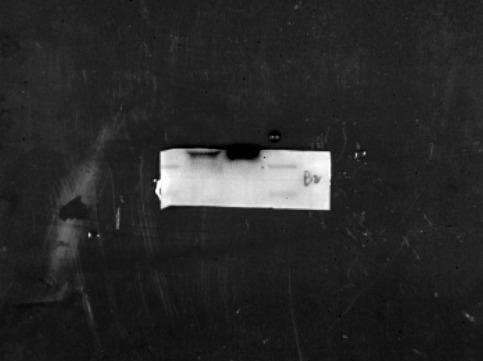

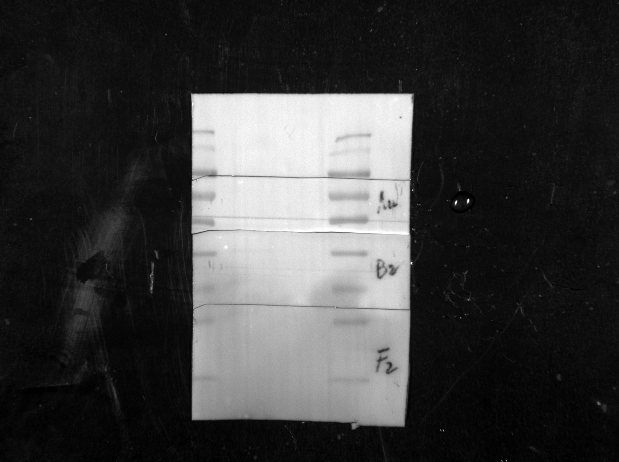


HSP70:


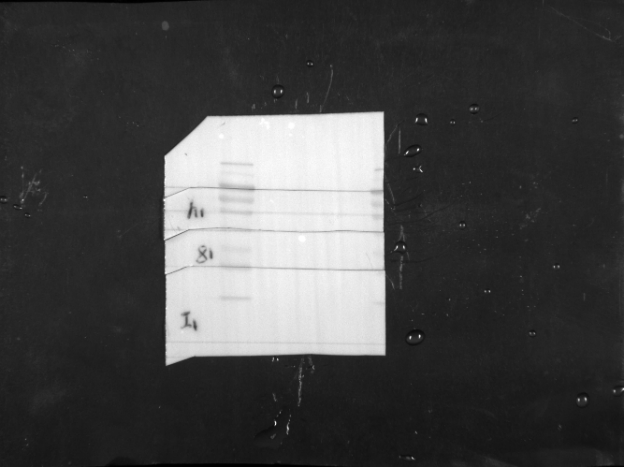


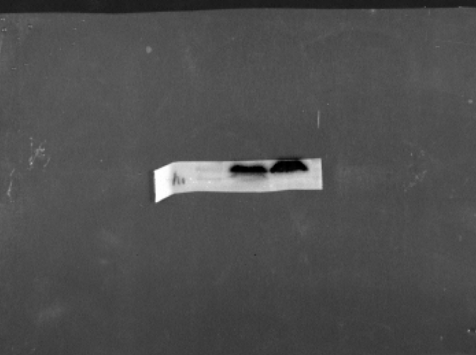


IGF2：


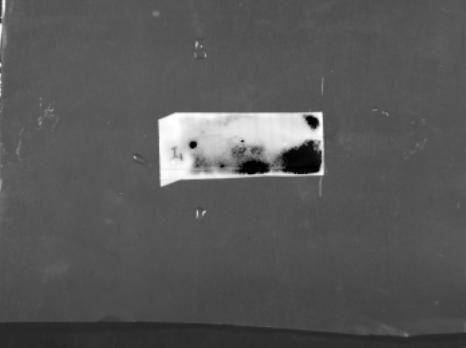


**3. Figure 3A：**

IGF-1R：


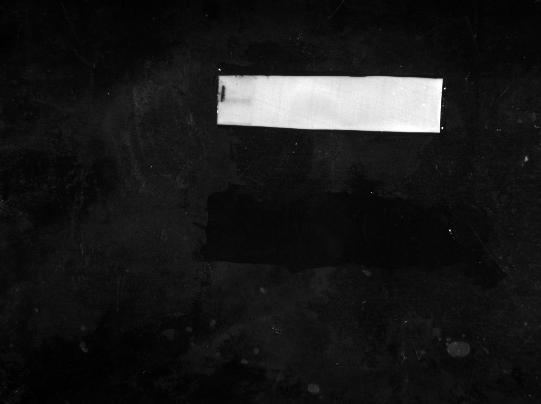

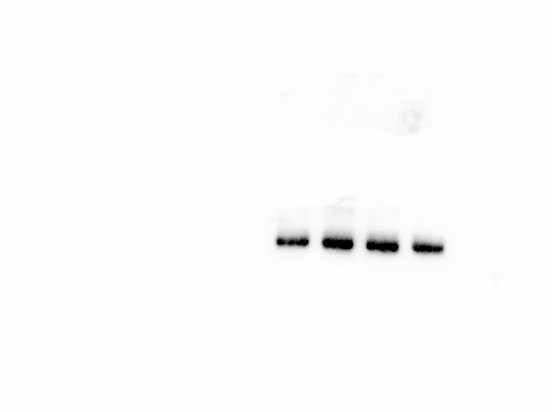


p-IGF-1R：


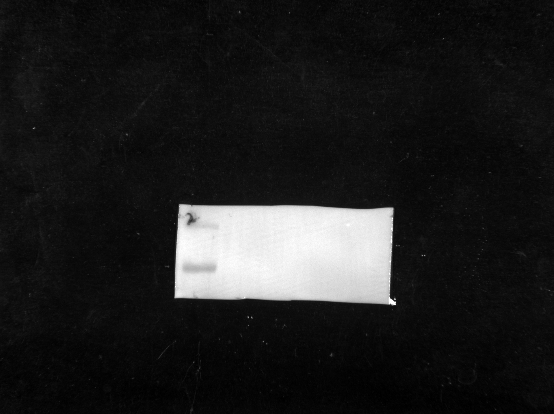

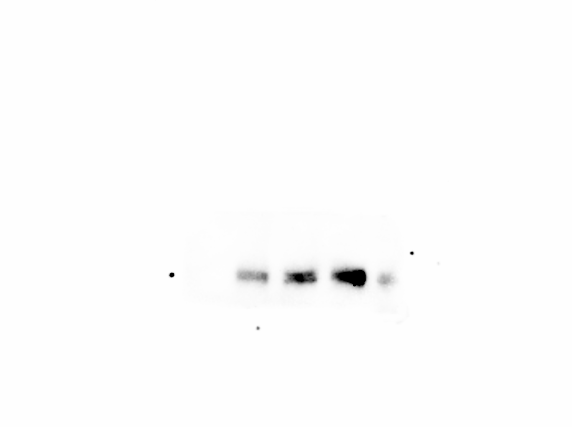


GAPDH：


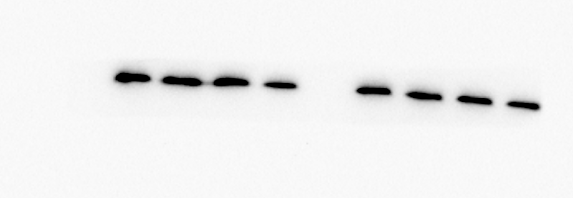

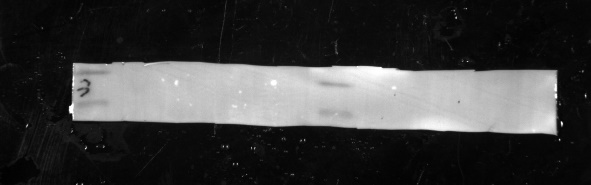

Supplement: Supplementary file 1 [file DataSheet_1.docx]
