# Supplementary material for: Exosomes from cisplatin-induced dormant cancer cells facilitate the formation of premetastatic niche in bone marrow through activating glycolysis of BMSCs
Source: Front Oncol. 2022 Dec 9;12:922465. doi: 10.3389/fonc.2022.922465 (PMC9786109; doi:10.3389/fonc.2022.922465)
Supplement: Supplementary file 2 [file DataSheet_2.docx]

Supplementary Material


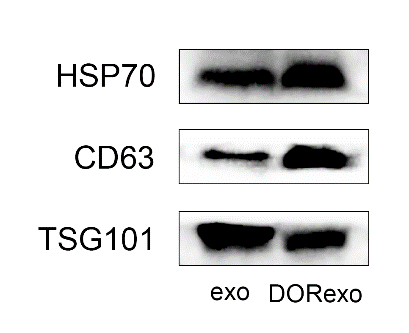


**Supplementary Figure 1.** Exosomal markers (HSP70, CD63, and TSG101) were analyzed by western blot. exo: exosomes from untreated A549 cells; DORexo: exosomes from dormant A549 cells (n=1).


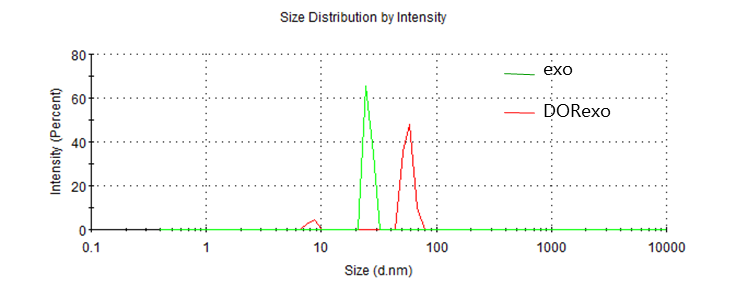


**Supplementary Figure 2.** Size distribution analysis of purified exosomes. exo: exosomes from untreated A549 cells; DORexo: exosomes from dormant A549 cells.


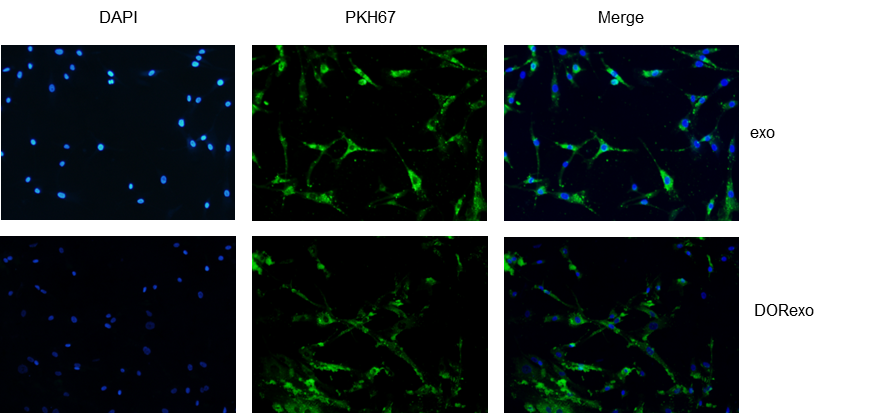


**Supplementary Figure 3.** PKH67 labeled exosomes entered into BMSCs after 6 hours’ incubation. DAPI was used to stain the nuclei. Representative images were filmed after cells were fixed and stained. exo: exosomes from untreated A549 cells; DORexo: exosomes from dormant A549 cells.


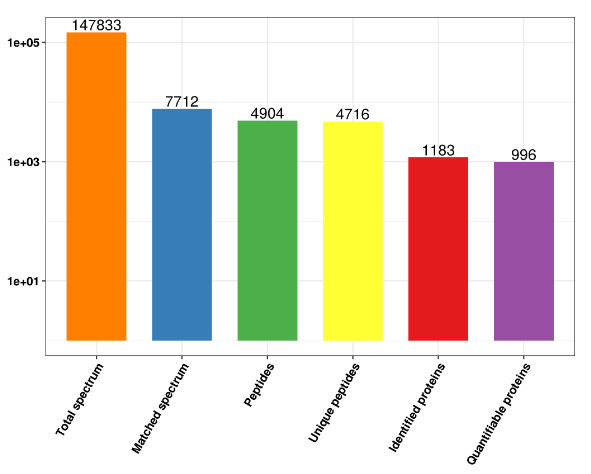


**Supplementary Figure 4.** Statistical graph of mass spectrum results. A total of 147833 secondary spectrograms were obtained by mass spectrometry analysis. The number of available spectra was 7712 and the utilization rate was 5.2%. A total of 4904 peptides were identified by spectrogram analysis, including 4716 specific peptides. A total of 1183 proteins were identified, of which 996 proteins could be quantified.


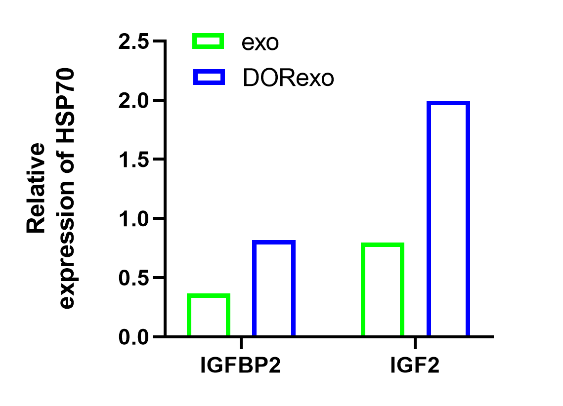


**Supplementary Figure 5.** Quantification of the band of western blot relative to the loading control (HSP70). The supplementary figure is corresponding to the Figure 2F (n=1).


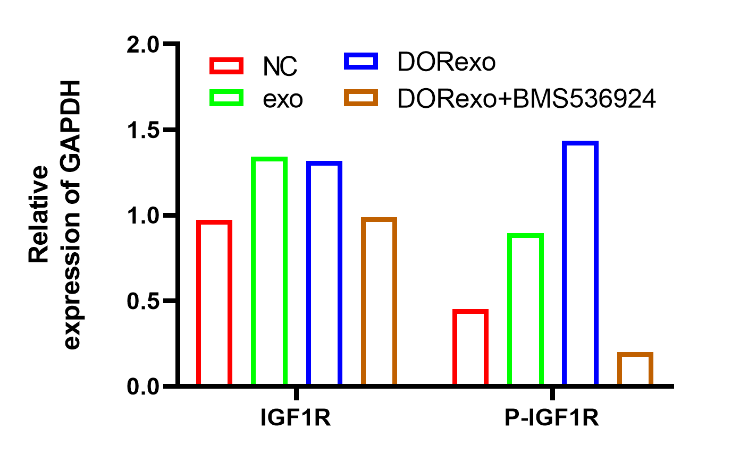


**Supplementary Figure 6.** Quantification of the band of western blot relative to the loading control (GAPDH). The supplementary figure is corresponding to the Figure 3A (n=1).


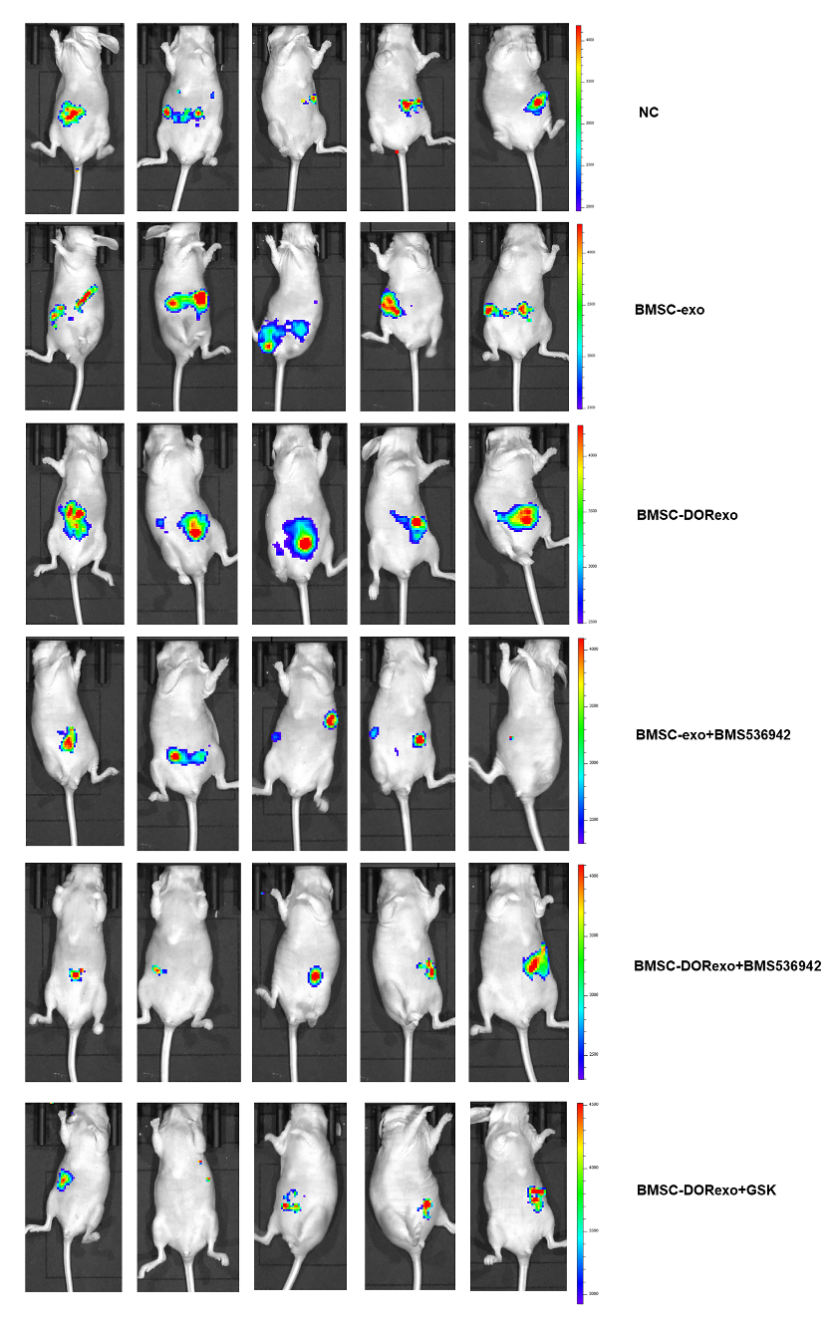


**Supplementary Figure 7.** Animal imaging fluorescence images on 63 days after A549-luciferase cells’ injection. NC: mice without BMSCs injection; BMSC-exo: mice with injection of exo-treated BMSCs; BMSC-DORexo: mice with injection of DORexo-treated BMSCs; BMSC-exo+BMS-536924: mice with injection of exo-treated BMSCs and BMS-536924; BMSC-DORexo+BMS-536924: mice with injection of DORexo-treated BMSCs and BMS-536924; MSC-DORexo +GSK :mice with injection of DORexo-treated BMSCs and GSK.
